# Progress in circRNA-Targeted Therapy in Experimental Parkinson’s Disease

**DOI:** 10.3390/pharmaceutics15082035

**Published:** 2023-07-28

**Authors:** Simoneide Souza Titze-de-Almeida, Ricardo Titze-de-Almeida

**Affiliations:** 1 Technology for Gene Therapy Laboratory, Central Institute of Sciences, University of Brasília, Brasília 70910-900, Brazil; 2 Research Center for Major Themes, Central Institute of Sciences, University of Brasília, Brasília 70910-900, Brazil

**Keywords:** circRNA, Parkinson’s disease, RNAi, neurodegenerative disease, oligonucleo-tide-based therapies

## Abstract

Circular RNAs (circRNAs) are single-stranded RNA molecules often circularized by backsplicing. Growing evidence implicates circRNAs in the underlying mechanisms of various diseases, such as Alzheimer’s and Parkinson’s disease (PD)—the first and second most prevalent neurodegenerative disorders. In this sense, circSNCA, circHIPK2, circHIPK3, and circSLC8A1 are circRNAs that have been related to the neurodegenerative process of PD. Gain-of-function and loss-of-function studies on circRNAs have shed light on their roles in the pathobiology of various diseases. Gain-of-function approaches typically employ viral or non-viral vectors that hyperexpress RNA sequences capable of circularizing to form the specific circRNA under investigation. In contrast, loss-of-function studies utilize CRISPR/Cas systems, antisense oligonucleotides (ASOs), or RNAi techniques to knock down the target circRNA. The role of aberrantly expressed circRNAs in brain pathology has raised a critical question: could circRNAs serve as viable targets for neuroprotective treatments? Translating any oligonucleotide-based therapy, including those targeting circRNAs, involves developing adequate brain delivery systems, minimizing off-target effects, and addressing the high costs of treatment. Nonetheless, RNAi-based FDA-approved drugs have entered the market, and circRNAs have attracted significant attention and investment from major pharmaceutical companies. Spanning from bench to bedside, circRNAs present a vast opportunity in biotechnology for oligonucleotide-based therapies designed to slow or even halt the progression of neurodegenerative diseases.

## 1. Introduction

Originally identified in viruses half a century ago [1], circular RNAs (circRNAs) were initially regarded as byproducts of aberrant splicing events, casting a cloud of uncertainty over their biological relevance until 2012. This viewpoint underwent a marked shift when circRNAs were found in archaea playing a specific biological function [2]. In recent years, due to advancements in RNA sequencing methodologies, an increased number of reports have delineated the structural architecture and functional roles of circRNAs in the metabolic machinery of various organisms, notably including humans [3,4,5].

The structure of circRNA comprises a circular single-stranded RNA molecule wherein the 3′ and 5′ ends are connected through a covalent phosphodiester bond [5,6]. This circular configuration predominantly arises from a process termed back-splicing of exons and/or introns [7,8]. The absence of free 3′ and 5′ ends, common targets of exonucleolytic degradation, confer to circRNAs exceptional stability, reflected in a half-life that surpasses 48 h [9]. This significantly contrasts with the approximately 10 h half-life of linear transcripts [10].

The eukaryotic circRNAs exhibit a unique expression pattern that is determined by tissue type, cell specificity, and developmental stage [9,11,12,13]. Particularly, they are abundant in the brain relative to other tissues and show sequence conservation from murine to human genomes [11,14,15]. Furthermore, circRNA expression is dynamically regulated during neuronal differentiation, nervous system development, and in response to synaptic stimuli [11,14]. Studies by Rybak-Wolf et al. (2015) demonstrated the compartment-specific expression of circRNAs within the brain, as various structures—including the olfactory bulb, prefrontal cortex, hippocampus, and cerebellum—produce distinct sets of highly expressed circRNAs [11]. Moreover, rather than a uniform distribution, circRNAs are strikingly concentrated within neuronal synapses, implying a potential role in the assembly of Ribonucleoprotein granules or in the transportation of proteins or RNAs. Consequently, circRNAs are proposed to function as regulatory structures that contribute to the intricate orchestration of gene expression during neuronal differentiation and function [11,14,16,17]. Importantly, numerous studies have underscored the significant involvement of circRNAs in the pathogenesis and progression of various diseases, including cancer and neurodegenerative disorders, such as Alzheimer’s and Parkinson’s disease (PD), respectively the first and second most prevalent neurodegenerative disorders [18,19,20].

## 2. Biogenesis and Functions of circRNAs

**Figure 1:**
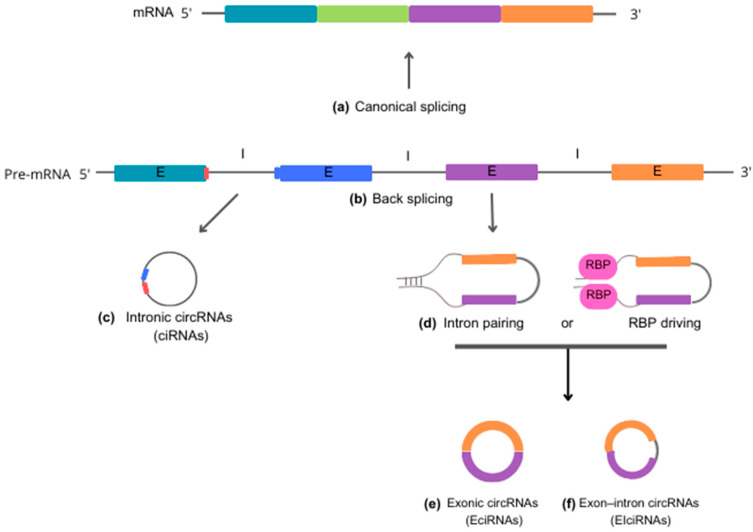
Schematic representation of circRNA biogenesis. Precursor mRNA (pre-mRNA) is the primary RNA transcript synthesized on a DNA template in the cell nucleus. Pre-mRNA can follow two distinct cellular pathways. In the upward direction, pre-mRNA gives rise to 5′-3′ mRNA sequences through canonical splicing (**a**), which removes introns and generates a molecule to be exported to the cytoplasm for guiding protein synthesis. In the alternate downward direction, pre-mRNA produces circRNAs through back-splicing reactions (**b**). Intronic circRNAs (ciRNA) (**c**) are formed by a lariat-driven cyclization process that exclusively involves introns. Specific sequence elements adjacent to an exon, rich in guanine (G) and uracil (U) (depicted in blue), bind to an 11-nucleotide cytosine (C)-rich sequence (shown in red) near another exon. This binding allows evasion of the debranching reaction and prevents degradation by exonucleases. Reverse complementary sequences and/or RNA-binding proteins (RBP) (**d**) act to bring the nucleotide sequences into close proximity. This process facilitates the back-splicing reaction, leading to the generation of exonic circRNA (EciRNA) (**e**) or exon-intron circRNA (EIciRNA) (**f**). Abbreviations: mRNA, messenger RNA; pre-mRNA, precursor mRNA; ciRNA, intronic circRNA; EciRNA, exonic circRNA; EIciRNA, exon-intron circRNA; E, exon; I, intron; RBP, RNA-binding proteins.

According to the molecular mechanisms underlying biogenesis, circRNAs can be classified into three primary categories: (i) intronic circRNAs (ciRNAs), (ii) exonic circRNAs (EcircRNAs), and (iii) exon-intron circRNAs (EiciRNAs). Each category represents a unique mode of circularization and has its own distinct biogenesis mechanisms [21] (Figure 1).

Intronic circular RNAs (ciRNAs) are generated through a lariat-driven cyclization process that involves only introns and takes place within the nucleus. In the standard splicing process, introns are typically excised from precursor RNA through splicing reactions and are ultimately degraded by exonucleases following a debranching reaction. However, in the case of ciRNAs, specific sequence elements adjacent to exons inhibit the removal of introns by the splicing machinery. These sequences, which are rich in guanine (G) and uracil (U) near one exon and feature an 11-nucleotide cytosine (C)-rich sequence near another exon, promote intron circularization. In this process, introns containing these unusual sequence elements evade debranching and instead form a circular structure, resulting in the formation of stable ciRNAs [6,22].

Exonic circRNAs (EcircRNAs) predominantly originate from one or multiple exons of pre-mRNA transcripts. They are abundant in the cytoplasm and may also be found in exosomes. The formation of EcircRNAs involves a process called intron-pairing-driven cyclization, in which complementary sequences within the same or different introns base-pair with each other, ultimately producing a circular RNA molecule composed entirely of exonic sequences. EcircRNAs represent a substantial proportion—more than 80%—of all circRNAs [21,23].

Exon-intron (EiciRNAs) are predominantly found in the nucleus, suggesting that they play a role in nuclear functions and processes. These particular circRNAs form as a result of intron retention between exons. The biosynthesis of EiciRNAs is driven by RNA-binding proteins (RBPs) that recognize and bind to specific gene sequences within the introns. This facilitates the creation of splicing sites at both ends of adjacent exons, which may occur through protein interactions or the formation of RBP dimers. Thereby, the interaction leads to the formation of covalent links between the splicing acceptor and donor sites, triggering the cyclization process and leading to the formation of EiciRNAs [8,24].

These three primary categories of circRNAs have been found to have specific functions and regulatory roles within cells. These roles can vary depending on the type and cellular context. Additionally, new categories of circRNAs have been identified, including fusion circRNAs (f-circRNAs), read-through circRNAs (rt-circRNAs), and mitochondria-encoded circRNAs (mecciRNAs) [21].

## 3. Function of circRNAs in the Cellular Metabolism

Although the understanding of the role of circular RNAs (circRNAs) in biological processes is still in its infancy, significant findings have been reported. CircRNAs may regulate gene expression by interacting with DNA (via interaction with RNA polymerase II or through methylation) and RNA (via mRNA trapping, acting as miRNA sponges, and influencing mRNA stability) [5,25]. Moreover, circRNAs can modulate protein interactions, act as protein sponges, and even synthesize short peptides or proteins [26,27,28].

## 4. CircRNAs Regulating Gene Expression—Interaction with DNA

### 4.1. Direct Regulation of Gene Expression

The majority of circRNAs are located in the cytoplasm, with a smaller portion found in the nucleus [29]. Within the nucleus, these circRNAs interact with RNA polymerase II at the promoter regions of host genes, thereby impacting transcription modulation [30].

### 4.2. Regulation of DNA Methylation

A recently discovered mechanism by which circRNAs modulate gene regulation involves altering the DNA methylation of downstream genes. For instance, circRNA ACR activates PTEN-induced putative kinase 1 (Pink1) expression by directly interacting with DNA methyltransferase 3 beta and inhibiting its role in DNA methylation at the Pink1 promoter [31].

### 4.3. Retrotransposon

Dong et al. (2016) have demonstrated the capacity of circRNA to form pseudogenes. While pseudogenes derived from linear mRNA maintain exon sequences consistent with their normal counterparts, those derived from circRNA possess exon connection sequences that are reversed in orientation compared to common genes. This finding suggests a potential role for circRNAs as reverse transposons, contributing to alterations in genome structure and the regulation of gene expression [32].

## 5. CircRNAs Regulating Gene Expression—Interaction with RNA

### 5.1. Regulation of Gene Expression

CircRNAs play a crucial role in the regulation of gene expression by actively influencing the transcription of linear RNA. Ashwal-Fluss et al. (2014) and Sinha et al. (2016) propose that when pre-mRNA contains a translation initiation site and undergoes nonlinear splicing, resulting in cyclization, it leads to a reduction in mRNA transcription. This decrease subsequently leads to a decline in downstream protein production for translation, a phenomenon known as the “mRNA trap” [7]. The competitive relationship between back-splicing and linear splicing may serve as a general mechanism for regulating mRNA processing connected to corresponding host genes [7,33].

### 5.2. Regulation of mRNA Stability

CircRNAs have been found to regulate mRNA stability in certain cases. Hansen et al. (2013) discovered that the circular RNA derived from the cerebellar-degeneration-related protein 1 (CDR1) gene forms a double-stranded structure with CDR1 mRNA, thereby enhancing its stability [34]. Additionally, in murine macrophages, Circ-Ras-GEF domain family member 1B (Ras-GEF1B) increases the stability of intercellular cell adhesion molecule-1 (ICAM-1) mRNA, leading to its heightened expression in the lipopolysaccharide (LPS)/toll-like receptor 4 (TLR4) inflammatory signaling pathway [35]. Garikipati et al. (2019) reported that CircFndc3b stabilizes the mRNA fused in sarcoma (FUS), as circFndc3b is primarily localized in the cytoplasm. These findings highlight the diverse roles circRNAs play in regulating gene expression [36].

### 5.3. Sponges of miRNAs

CircRNAs possess specific interaction sites, referred to as miRNA response elements (MREs), which enable them to function as sponges for miRNAs, drawing miRNAs away from their target mRNAs and thereby influencing gene expression and protein synthesis. This mechanism is one of the ways circRNAs can regulate cellular functions and contribute to physiological and pathological processes. Li et al. (2023) demonstrated that a single circRNA might have multiple and efficient miRNA-binding sites, conferring upon them a significant regulatory role in miRNA biology [37].

## 6. CircRNAs Regulating Cellular Metabolism—Interaction with Protein

### 6.1. Protein Translation

Gene translation into proteins typically necessitates the recognition of the 5′ cap structure of mRNAs [38]. However, recent studies have revealed that circRNAs can also be translated into proteins, given the presence of internal ribosome entry site (IRES) elements within their structure [26]. The initial discovery of a circRNA functioning as a protein translator was reported in the hepatitis C virus; this single-stranded circRNA produced a protein consisting of 122 amino acids [39].

The circRNAs, with their versatile regulatory capacity, significantly contribute to orchestrating the intricate network of cellular metabolism. These molecules play a crucial role in cellular function, particularly in brain tissue. Thus, exploring the role of circRNAs in neurodegenerative diseases, such as PD, could bring new therapeutic strategies to interrupt disease progression [26,40].

### 6.2. Sponges and Proteins

In addition to MREs, circRNAs also possess protein interaction sites within their structure. Recent research by Ulshöfer et al. (2021) and Aufiero et al. (2019) has found that these sites function as protein sponges, impacting protein expression, biogenesis, and pathophysiological progression. This expands our understanding of the multifaceted regulatory capabilities of circRNAs in gene expression and their influence on cellular processes [41,42].

## 7. CircRNAs Relationship between Alpha-Synuclein and microRNAs in the Parkinson’s Disease

**Figure 2:**
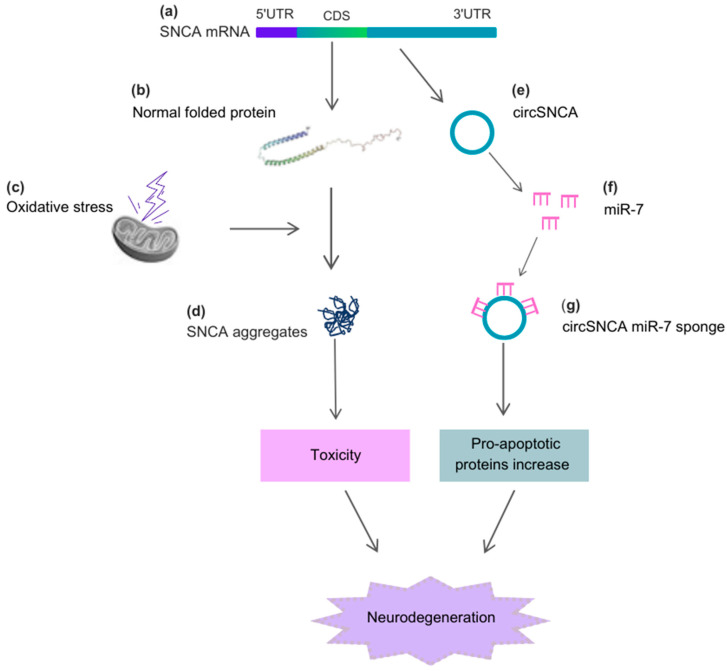
Alpha-synuclein (SNCA), circSNCA, and the cellular stressors acting on the neurodegenerative process of PD. (**a**) The CDS region of SNCA mRNA originates the (**b**) normal folded SNCA protein, which, under (**c**) oxidative stress, causes the formation of (**d**) SNCA aggregates. These aggregates induce toxicity and further neurodegeneration. The 3′UTR region of SNCA mRNA originates (**e**) circSNCA, which acts as a sponge for miR-7 (**f**,**g**), leading to an increase in pro-apoptotic proteins and neurodegeneration. Abbreviations: 5′UTR, 5′untranslated region; 3′UTR, 3′untranslated region; CDS, coding sequence.

Alpha-synuclein (SNCA) is a cytosolic protein that is abundantly expressed in a healthy brain and plays crucial roles in neurotransmission through interactions with synaptic vesicles and proteins involved in exocytosis [43]. In cells, SNCA functions by regulating protein ubiquitination, chaperone activity, kinase-dependent pathways, and dopamine metabolism, which is a neurotransmitter depleted in Parkinson’s disease (PD) [44]. Exposure to cellular stressors triggers structural changes in SNCA, leading to the formation of fibrillar aggregates called Lewy Bodies and Lewy neurites [45]. These SNCA inclusions, prominently observed in nigral dopaminergic neurons, are hallmarks of both sporadic and familial forms of PD [46,47,48]. Circular RNA (circRNA) can also be derived from the proximal 3′UTR of SNCA mRNA, containing the high-affinity site for miR-7, and can efficiently sponge miR-7 in cell culture [49]. When human neuroblastoma SH-SY5Y cells are treated with the dopamine agonist pramipexole, circSNCA content decreases, leading to increased miR-7 levels and a subsequent decrease in SNCA protein levels. Furthermore, higher circSNCA expression has been reported to be associated with increased expression of pro-apoptotic proteins such as CASP3, BAX, PTEN, and P53, as well as decreased content of autophagy-associated protein LC3B-II [49] (Figure 2).

One of the best-characterized circular RNAs is the Cerebellar Degeneration-Related Protein 1 Antisense RNA (CDR1as), which is highly expressed in the mammalian brain. It is part of a regulatory network of non-coding RNAs, including miR-7, miR-671, and the cyrano-long RNA [34,50]. CDR1as contains over 70 binding sites for miR-7 [34]. Recent research indicates that knockout of CDR1as results in miR-7 depletion, associated with synaptic dysfunction and significant sensorimotor alterations [51]. CDR1as has a site that tightly binds miR-671, exhibiting nearly perfect complementarity to the mature sequence of the microRNA. This interaction allows miR-671 to induce CDR1as cleavage, catalyzed by Argonaute 2 [34,51]. As proposed by Piwecka et al. (2017), in terms of regulation, CDR1as manages miR-7 levels, while miR-671 controls cellular CDR1as content [51].

In a model of cancer, by using a biotinylated circHIPK3 probe, Zeng et al. (2018) showed that miR-7 was pulled down by circHIPK3. These findings were corroborated with additional assays, and ectopic expression of circHIPK3 reversed miR-7 inhibition of its targets [52].

Circzip-2, one of the two most highly expressed circRNAs in *C. elegans*, was observed to be downregulated 18-fold in the SNCA-overexpression PD model when compared to the wild-type strain of *C. elegans*. This circRNA potentially functions as a sponge for miR-60-3p, which suppresses mRNAs involved in the forkhead box O (FOXO) pathway. This pathway plays a protective role in the development of PD and aging [53].

A recent study discovered 24 circRNAs with differential expression in substantia nigra (SN), medial temporal gyrus, and amygdala tissue samples from Parkinson’s disease (PD) patients. For instance, circSLC8A1 was found to be increased in the SN of PD patients. It contains seven binding sites for miR-128, which also show elevated levels in PD patients, suggesting a potential involvement of circSLC8A1 in regulating miR-128 function and/or activity. Furthermore, circSLC8A1 levels exhibited higher expression in cultured cells exposed to the oxidative stress-inducing agent paraquat, while its levels decreased following treatment with a neuroprotective antioxidant agent. This indicates a possible connection between circSLC8A1 and oxidative stress-related parkinsonism [54]. Another study using a mice model intraperitoneally injected with 1-methyl-4-phenyl-1,2,3,6-tetrahydropyridine (MPTP) identified differentially expressed circRNAs related to PD in the mice cerebellum through RNA-seq analysis [55].

## 8. CircRNA in Neuroinflammation

A thorough study of the circRNA transcriptome in human brain glial cells, including astrocytes, microglia, and oligodendrocytes, highlights the unique nature of each transcriptome, pointing to their specific roles within the brain. The researchers found 265, 239, and 442 unique circRNAs in astrocytes, microglia, and oligodendrocytes, respectively. Intriguingly, the highest prevalence of circRNAs in these glial cell types comes from parent genes that predominantly express linear RNAs at relatively low levels, hinting at a preference for spliceosome activity targeting the back-splicing mechanism over the canonical splicing activity [56].

CircPtk2, associated with neuroinflammation, has been found to act as a sponge for miR-29b in a cerebral ischemia model [57]. When microglial culture-derived conditioned media were introduced to hippocampal neurons, apoptosis resulted but was alleviated by prior miR-29b overexpression in microglia. Changes in the expression of the miR-29 family were previously identified in the blood serum of patients with PD compared to a control group of healthy individuals (*n* = 80 for each group). The findings revealed a significant decrease in components of the miR-29 family with increasing disease severity [58].

CircRNAs are also linked to astrocytic activation [59,60]. Studies indicate that methamphetamine-induced degeneration of dopaminergic neurons in the SNpc corresponds with widespread reactive astrogliosis in the striatum [61]. By administering siRNA or lentiviral shRNA targeting circHIPK2, methamphetamine-induced astrocytic activation is noticeably suppressed due to the decrease of Sigma non-opioid intracellular receptor 1 (SIGMAR1) expression. SIGMAR1 is critical to astrocytic activation in vitro and in vivo [59,60] and is a target of miR-124 [60]. circHIPK2 acts as an endogenous sponge for miR-124. Decreased expression of miR-124 is observed in PD patients [62,63], and overexpression in PD mice leads to enhanced motor functions, reduced dopaminergic neuron loss, and diminished oxidative stress [63]. Corroborating this notion, Yao et al. (2018) showed that miR-124 can suppress neuroinflammation in the MPTP-induced model of Parkinsonism [64]. Recently, Zhang et al. (2023) showed that the expression of circHIPK3 in human serum and cerebral fluids of PD patients was significantly higher than in controls, followed by a significant reduction of miR-124 expression. The study also found that lipopolysaccharide (LPS)-treated BV2 cells exhibited higher expression of circHIPK3 and lower miR-124 expression. In addition, by using SH-SY5Y cells, these authors showed significantly impaired viability and elevated apoptotic rate, along with an upregulation of circHIPK3 and downregulation of miR-124 expression after treatment with conditionate medium (LPS-treated BV2 cells). The circHIPK3 enhances neuroinflammation by sponging miR-124 and regulating the miR-124-mediated STAT3/NRLP3 pathway in PD [62].

## 9. Scientific Methodologies to Target circRNAs

The scientific study of circRNAs typically involves suppressing the target and evaluating the resultant biological response in such experimental preparations. Additionally, researchers may overexpress a circRNA of interest to assess whether the change can ameliorate a pathological process in analysis, for example [26]. In this section, we will outline several strategies commonly used for silencing or overexpressing specific circRNAs, also called loss-of-function or gain-of-function approaches.

### 9.1. Strategies to Downregulate circRNAs

#### 9.1.1. Synthetic Small-Interfering RNAs (siRNAs)

The discovery of RNA interference (RNAi) in 1998 by Nobel laureates Fire and Mello has had a significant impact on the field of cell biology [65]. Among the most valuable applications of RNAi technology is the use of small-interfering RNAs (siRNAs) to selectively silence specific molecular targets, including circular RNAs of interest [26].

**Figure 3:**
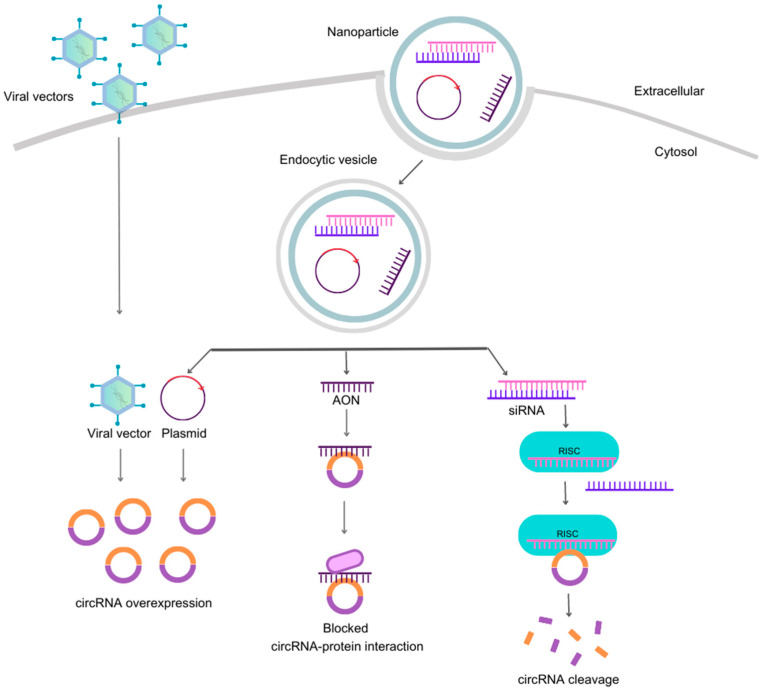
Strategies for targeting circRNAs. The four most common methods to modify circRNA content include viral and non-viral vectors, AONs, and small-interfering RNAs (siRNAs). On the left, engineered viral vectors with specific cassettes enter the cell without carriers and overexpress a specific circRNA. The other strategies for targeting circRNAs require a nanoparticle to carry nucleic acids (siRNA, AON and plasmid) across the plasma membrane into the cytosol, which then form endocytic vesicles. Upon vesicle disruption, the plasmids or oligonucleotides are free to perform their functions. Plasmids are circular double-stranded DNA sequences engineered to overexpress single-stranded RNA that can circularize and form the intended circRNA, providing gain-of-function effects similar to viral vectors. In contrast, loss-of-function effects are achieved with AONs, single-stranded RNA sequences that bind through Watson–Crick base pairing to complementary nucleotides in circRNA, blocking protein interaction sites. Additionally, siRNAs are short double-stranded RNA molecules that trigger RNAi mechanisms involving the RISC complex, leading to circRNA cleavage. Abbreviations: circRNA, circular RNA; siRNA, small-interfering RNA; RISC, RNA-inducing silencing complex.

siRNAs are short RNA molecules that identify a target RNA by Watson–Crick base-pairing for cleavage aided by RNAi proteins [66]. RNAi-mediated gene silencing by siRNAs occurs through a cellular process, as illustrated in Figure 3 and described in further detail elsewhere [67]. Typically, synthetic siRNA are duplexes containing 19–23 ribonucleotides. The duplexes are loaded onto a protein complex called the RNA-induced silencing complex (RISC), which removes the passenger strand and uses the remaining guide strand to search for complementary nucleotides on target RNA molecules. Once a match is found, the guide strand-attached RISC cleaves the targeted RNA, leading to gene silencing [68]. This approach can also be applied to circRNAs targeting the back-splice junction in order to avoid silencing of the respective linear mRNA [26].

Synthetic siRNAs are readily available from many suppliers and can now be obtained with incorporated structural changes that enhance their specificity and durability. In typical cell culture experiments, siRNA molecules are frequently complexed with lipid or polymeric particles to ensure intracellular delivery [69]. In vivo administration of siRNAs, on the other hand, requires careful selection of delivery particles from a range of commercially available options, some of which have received FDA approval for siRNA-based therapeutics [70,71]. In a specific section below, we present examples of siRNAs utilized to downregulate circRNAs in studies focused on neurodegeneration.

#### 9.1.2. Short-Hairpin RNAi Expressing Vectors

Expressing vectors are exogenous systems that utilize the cell machinery to express RNAi duplexes. These vectors enable the expression of an RNA strand that folds and forms a duplex of paired nucleotides, as well as a loop of unpaired nucleotides at one end of the molecule, thus referred to as short hairpin duplexes. Expression vectors are typically categorized as non-viral expressing vectors, such as plasmids and viral expressing vectors [72,73].

#### 9.1.3. Antisense Oligonucleotides

Antisense oligodeoxynucleotides (AONs) are single-stranded molecules that bind to target mRNAs with complementary sequences, facilitating their degradation through endonuclease activity [74]. Similar to small interfering RNAs (siRNAs), AONs have undergone chemical modifications to enhance specificity and stability, including phosphorothioate backbones and locked nucleic acids [75]. Recent advancements in carrier technologies have improved the delivery of oligonucleotides, enabling several AONs to enter the pharmaceutical market [76,77]. In addition, AONs provide a method to silence circRNAs [78] or to block protein interaction sites within circRNAs [26].

#### 9.1.4. CRISPR/Cas Systems for circRNA Knockout or Knockdown

The Clustered Regularly Interspaced Short Palindromic Repeats (CRISPR) system is an efficient and precise genome-editing technique often used for the knockdown or knockout of specific genomic regions of interest in order to investigate their biological functions [79]. This method eliminates the labor-intensive process of creating transgenic mice, thereby significantly speeding up scientific research. The CRISPR/Cas9 process involves the Cas9 endonuclease and requires the use of accessory RNA molecules, as described in prior published reviews [80,81]. In recent years, CRISPR systems and their associated Cas proteins have experienced substantial advancements across various fields of knowledge, including neuroscience [82]. This progress has facilitated the development of innovative research methodologies and therapeutic applications [83].

### 9.2. Overexpression of circRNAs by Nonviral and Viral Vectors

In gain-of-function studies, various vectors have been investigated for overexpressing circular RNAs (circRNAs) [26,40]. Plasmids harboring circRNA-producing exons and their respective adjacent intronic sequences are extensively used [84]. Additionally, viral vectors provide an alternative approach for circRNA overexpression; for instance, adenoviral- and lentiviral vectors equipped with intron-containing cassettes have been shown to promote back-splicing, leading to circRNA formation [40,85,86]. Importantly, a previous study devised and evaluated multiple circRNA expression techniques, ultimately identifying an efficient transposon-mediated overexpression system [87]. The subsequent topic presents examples of vectors employed for circRNA overexpression in experimental models of neurodegeneration.

### 9.3. Investigating the Role of circRNAs in Models of Neurodegenerative Diseases: siRNAs and Expression Vectors in Selected Studies

As previously highlighted, researchers commonly manipulate circRNA levels using small interfering RNAs (siRNAs) for downregulation and expression vectors for upregulation, with the aim of investigating the roles of specific circRNAs in disease pathogenesis.

Feng et al. (2020) examined the role of circular RNA DLGAP4 (circDLGAP4) in MPTP-induced mice model of Parkinson’s disease and MPP^+^ (1-methyl-4-phenylpyridinium) treated SH-SY5Y cells. Additionally, the researchers investigated the circDLGAP4 in MN9D, a hybrid mouse dopaminergic neuronal cell line. In these Parkinson’s disease models, injury resulted in the downregulation of circDLGAP4. Likewise, siRNA-mediated knockdown of circDLGAP4 in SH-SY5Y and MN9D cells led to a decrease in cell viability, an increase in apoptosis, and mitochondrial damage. In contrast, overexpression of circDLGAP4 using a circDLGAP4 expression plasmid reversed the damage induced by MPP+ in both cell lines. The researchers suggested that the neuroprotective effects of circDLGAP4 were mediated through modulation of the miR-134-5p/CREB pathway [88].

The study conducted by Feng’s group corroborated the neuroprotective role of circDLGAP4 first proposed in another brain disease: stroke [89]. This study found circDLGAP4 downregulated in the plasma of patients with acute ischemic stroke, as well as in mice subjected to a transient middle cerebral artery occlusion stroke model. The authors explored whether circDLGAP4 would exhibit neuroprotective effects post-stroke. To this end, circDLGAP4 was overexpressed via lentiviral injection into the lateral ventricles of animals in the stroke model. Mice with increased circDLGAP4 expression exhibited reduced neurological deficits, smaller infarct areas, and better preservation of the blood-brain barrier compared to the control group. Additionally, the authors demonstrated that circDLGAP4 acts as an endogenous miR-143 sponge, which inhibits miR-143 activity [89].

Hanan et al. (2020) utilized synthetic siRNAs to selectively silence circSLC8A1 in order to examine its potential role in modulating miR-128 activity [54]. The researchers initially observed elevated circSLC8A1 levels in the substantia nigra of Parkinson’s disease patients and identified circRNA binding sites for both miR-128 and the microRNA effector protein Ago2. Upon exposure to paraquat-induced oxidative stress, circSLC8A1 levels increased in SH-SY5Y cultured cells. To further investigate the effects of silencing this circRNA on mRNA expression, cell transfection with siRNAs targeting circSLC8A1 was performed, followed by RNA-seq analysis. Notably, several upregulated genes were identified as miR-128 targets. By employing a combination of siRNA-mediated circSLC8A1 knockdown and bioinformatic analysis, the authors provided evidence supporting their hypothesis that circSLC8A1 plays a role in modulating miR-128 function [54].

Additional studies have also utilized synthetic siRNAs and expression vectors to investigate the roles of specific circRNAs in mice models of Parkinsonism and dopaminergic cells, including circRNA derived from the pantothenate kinase 1 (Pank1) gene (circ-Pank1) and circSV2b [90,91].

#### 9.3.1. Paving the Path for circRNA Development: Drawing on the Success of siRNAs in Reaching the Market

The translation of noncoding RNAs from bench to clinic has generally proceeded at a satisfactory pace, as evidenced by the experience with small-interfering RNA (siRNA) [70,71]. Following the discovery of gene silencing in *C. elegans*, the first siRNA, patisiran, received FDA approval just two decades later, in 2018 [65,92]. While siRNAs represent a relatively new drug class, circular RNAs (circRNAs) are still in their infancy, as mentioned earlier. Nevertheless, circRNAs are promising drug targets that warrant increased attention from the scientific community and the pharmaceutical industry [26,40].

siRNAs are used exclusively for gene silencing, with the aim of downregulating a pathogenic protein responsible for a specific disease, such as the transthyretin proteins in patisiran or delta-ALA synthase 1 (ALAS1) enzyme in givosiran [92,93]. circRNA technology may employ siRNAs for silencing overexpressed circRNAs associated with neuropathology. In this context, siRNA-targeting circRNAs could leverage previous technological advancements in platforms tailored for siRNA-based drugs that have successfully passed Phases I–III of clinical testing and entered the market. These technologies involve chemical modifications in oligonucleotide molecules to generate more stable and target-specific drugs, as well as the development of improved carriers for siRNA delivery [70,76].

In the search for innovative therapeutic approaches for brain injection, nanocarriers, such as Neuromag, have emerged as promising candidates. Neuromag, a magnetic particle, has been successfully administered into the lateral ventricle of the rat brain to deliver oligonucleotides to the striatum via magnetofection. This process leads to the downregulation of a specific target miRNA [94]. The technique holds the potential for adapting to manipulate aberrantly expressed circRNAs in the striatum, a region critically involved in Parkinson’s disease neuropathology.

In some instances, neuropathology is not attributable to the overexpression of circRNAs; rather, it is the downregulation that poses a problem. This necessitates the exogenous restoration of low levels of circRNAs. Viral vectors provide a stable mechanism for the overexpression of oligonucleotides and can be injected into specific brain regions to compensate for the endogenous production of circRNAs. The technique of injecting viral vectors into the human brain has been extensively explored for the delivery of the trophic factor neurturin via an adeno-associated type-2 vector (AAV2) AAV2-NRT [95,96]. Importantly, prior studies on animal models of Parkinson’s disease have demonstrated that intranigral injections of viral vectors targeting circRNAs, equipped with well-constructed cassettes and methodologies for expressing circularizable RNA sequences, can increase endogenous circRNA levels and alleviate brain damage and behavioral deficits [90,91].

In pharmacology, the common approach is to search for drugs that act with high affinity on specific targets in order to achieve the desired effect. For instance, propranolol is an antagonist of all beta-adrenergic receptors, whereas metoprolol selectively blocks the beta-1 subtype of adrenergic receptors, providing a cardioselective effect. This concept is notably applicable to siRNAs, which target a specific mRNA. However, it is not relevant for miRNAs, which bind to hundreds of targets. Interestingly, experimental effects can be achieved using either siRNAs or miRNAs, suggesting that the number of drug target specificity in oligonucleotide therapy is not a prerequisite for drug efficacy [46,97].

In the context of circRNAs, siRNA molecules can be utilized to selectively target specific sequences within the circularized RNA under investigation, thereby initiating post-transcriptional gene silencing. However, a single circRNA often has multiple binding sites for microRNAs (each microRNA downregulates hundreds of mRNA targets) and proteins. Consequently, the knockout of specific circRNA may result in substantial changes in the cell transcriptome, as demonstrated in a previous study with CDR1as [51]. In a similar manner, overexpression of a particular circRNA may alter the function of numerous cellular targets. It can be argued that a more widely distributed cell effect may be beneficial for protecting or restoring cell biology under injury, considering that damaged or even apoptotic cells initiate multiple signaling pathways.

#### 9.3.2. Are circRNAs Gaining Traction in the Pharmaceutical Market?

The potential of circular RNAs (circRNAs) as drug targets has recently garnered significant attention from the pharmaceutical industry. This enthusiasm is evidenced by the substantial investments made towards the development of circRNA-targeted therapeutics, which show promise in addressing a variety of illnesses, such as cancer, cardiovascular diseases, and neurodegenerative disorders, such as Alzheimer’s and Parkinson’s [26,98,99]. A prominent example is Merck’s announcement of an initial $150 million investment in oRNA Therapeutics to bolster this emerging field, with the possibility of expanding to as much as $3.5 billion for continued development of vaccines and therapeutic products targeting infectious diseases and oncology [100].

## 10. Conclusions

The major challenges associated with developing circRNA-targeted therapeutics for neurodegenerative disorders involve ensuring efficient brain delivery, minimizing off-target effects, and addressing financial constraints [26,40,84,101]. Despite these ongoing concerns, oligonucleotide-based therapies, specifically RNAi-based treatments, have achieved FDA approval and entered the market as pharmaceutical drugs. These therapies have successfully advanced past the “proof-of-concept” stage, undergone clinical trials, and ultimately been integrated into patient care [71,102].

The use of synthetic siRNAs, which target overexpressed pathogenic circRNAs, is considered a promising approach for circRNA-targeted therapy in neurodegenerative diseases [26]. Previous studies have shown that circSNCA-targeted siRNAs can effectively silence the overexpression of alpha-synuclein and proapoptotic genes induced by MPP+ in SH-SY5Y cells. Additionally, the use of these siRNAs prevents the downregulation of miR-7 [49]. Regarding the administration route for circRNA-targeted therapy, both the striatum and lateral ventricle have been identified as suitable sites for brain stereotaxic injections, using either RNA oligonucleotides complexed with particles or viral vectors. The effectiveness of these administration routes has been demonstrated in previous studies involving animal models or patients with PD [89,94,103,104,105].

In conclusion, the emerging field of circRNA-targeted biotechnology shows significant potential, as supported by recent data. The technical approaches being employed share similarities with those used in FDA-approved medications based on RNAi. Based on the observed progress, it is evident that circRNA-targeted biotechnology offers promising prospects for the development of innovative therapeutics in the near future.

## Data Availability

Not applicable.
